# Current status and perspective of CAR-T and CAR-NK cell therapy trials in Germany

**DOI:** 10.1038/s41434-021-00246-w

**Published:** 2021-03-22

**Authors:** Nawid Albinger, Jessica Hartmann, Evelyn Ullrich

**Affiliations:** 1 Children’s Hospital, Division for Stem Cell Transplantation, Immunology and Intensive Care Medicine, Goethe-University Frankfurt, Frankfurt am Main, Germany; 2 Experimental Immunology, Goethe University Frankfurt, Frankfurt am Main, Germany; 3 Division of Medical Biotechnology, Paul-Ehrlich-Institut, Langen, Germany; 4 Frankfurt Cancer Institute, Goethe University, Frankfurt am Main, Germany; 5 German Cancer Consortium (DKTK), Partner Site Frankfurt/Mainz, Frankfurt am Main, Germany

**Keywords:** Cancer, Immunotherapy

## Abstract

Chimeric antigen receptor (CAR)-T cell therapies are on the verge of becoming powerful immunotherapeutic tools for combating hematological diseases confronted with pressing medical needs. Lately, CAR-NK cell therapies have also come into focus as novel therapeutic options to address hurdles related to CAR-T cell therapies, such as therapy-induced side effects. Currently, more than 500 CAR-T and 17 CAR-NK cell trials are being conducted worldwide including the four CAR-T cell products Kymriah, Yescarta, Tecartus and Breyanzi, which are already available on the market. Most CAR-T cell-based gene therapy products that are under clinical evaluation consist of autologous enriched T cells, whereas CAR-NK cell-based approaches can be generated from allogeneic donors. Besides modification based on a second-generation CAR, more advanced CAR-immune cell therapeutics are being tested, which utilize precise insertion of genes to circumvent graft-versus-host disease (GvHD) or employ a dual targeting approach and adapter CARs in order to avoid therapy resistance caused by antigen loss. In this review, we are going to take a closer look at the commercial CAR-T cell therapies, as well as on CAR-T and CAR-NK cell products, which are currently under evaluation in clinical trials, that are being conducted in Germany.

## Background on chimeric antigen receptor (CAR) therapy

For decades the only available cancer treatments were surgical resection as well as chemotherapy and/or radiotherapy [1]. However in the last years, immunotherapies using immune-checkpoint inhibitors (ICI), as well as cellular therapies, have moved into the limelight as promising alternative treatments. Normally, potentially malignant cells are continuously eliminated by the immune system, but cancer cells can accumulate certain mutations, which allow them to escape these mechanisms [2]. Cancer immunotherapies aim to support or boost the patient’s immune system to enable the effective clearance of cancer cells.

One way to achieve this is to genetically modify immune cells, mainly T cells and recently also natural killer (NK) cells, to express chimeric antigen receptors (CARs). CAR-expression on T or NK cells allows them to specifically target cancer cells via recognition of tumor associated antigens. “*Classical*” CARs consist of an extracellular binding domain mostly derived from a monoclonal antibody fragment (single-chain variable fragment-scFv), which is linked to intracellular binding domains of the T-cell receptor complex. Binding of a tumor antigen via the scFv activates the T cell in a major histocompatibility-independent manner which leads to a cytotoxic response [3]. Novel CAR constructs are being continuously developed, which can possess altered intracellular co-stimulatory domains and/or targeting domains. Latter can consist of different molecules, such as nanobodies, designed ankyrin repeat proteins (DARPins), ligands, or receptors instead of scFvs [4–7]. Furthermore, the so-called adapter CARs have been developed by splitting antigen recognition and CAR-immune cell activation. The addition of separate adapter molecules (AMs) specific for tumor antigens and CAR-immune cells targeting these AMs allows a more precise and temporally limited therapy. Thereby, several antigens can be targeted at once and the therapy can be adapted in case antigen-loss tumor variants appear. Additionally, this approach opens the possibility to shut down the immune response if severe side effects emerge [8, 9].

**Fig. 1:**
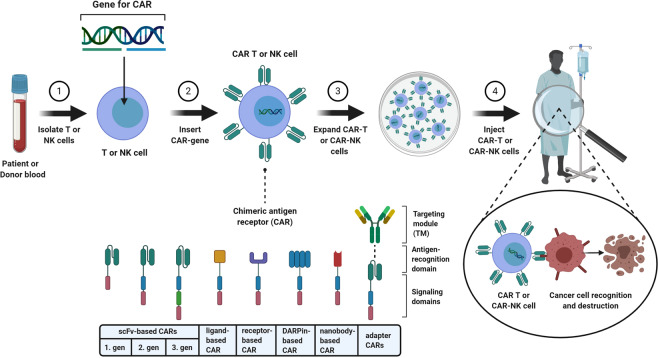
Schematic illustration of a CAR-T or CAR-NK cell therapy, which uses primary immune cells. (1) T or NK cells are isolated from the patient’s or donor’s blood. (2) Subsequently, cells are genetically modified to express chimeric antigen receptors (CARs). (3) CAR-T or CAR-NK cells are expanded until sufficient cell numbers are attained and (4) (re-)injected into the patient’s body, where they can fight cancer cells. CAR constructs possess a targeting module that recognizes tumor antigens and either a single intracellular signaling domain (1. gen) or, one (2. gen), or two (3. gen) additional co-stimulatory domains. In most CAR constructs the targeting module consist of a single-chain variable fragment (scFv). Novel CAR constructs can also possess nanobodies, designed ankyrin repeat proteins (DARPins), ligands, or receptors instead of scFvs for target recognition. Adapter CARs consist of two components: a soluble antigen targeting module (TM) and a CAR which targets this TM. CAR chimeric antigen receptor, DARPins designed ankyrin repeat proteins, gen generation, scFv single-chain variable fragment, TM targeting module. *This figure has been created using BioRender*.

An autologous CAR-T or NK cell therapy comprises several steps as shown in Fig. 1. First, T or NK cells are isolated from patient’s or donor’s blood. Subsequently, cells are transduced with CAR-encoding genes using (mostly) viral vectors. CAR-modified immune cells are expanded until sufficient cell numbers are attained and are adoptively transferred into the patient to fight malignant cells. Prior to infusion of the CAR-modified immune cells, lymphodepletion is performed in most therapeutic settings to allow efficient cell engraftment [10]. It is important to mention that, CAR-NK cells offer the potential to be an “off-the-shelf” product, but also allogeneic CAR-T cell therapies are currently under development [11–14].

## Overview of CAR-T and CAR-NK cell therapies worldwide

CAR-T cells have shown astonishing results in the treatment of mostly relapsed or refractory (r/r) hematological malignancies, which have already resulted in the approval of three drugs by the *U.S. Food and Drug Administration* (FDA) and the European Commission (EC) between 2017 and beginning of 2021 [15–17]. Currently, a fourth CAR-T cell product is under evaluation in Europe, which has been approved by the FDA lately [18]. Based on these crowning achievements, many CAR-T cell therapies are currently under evaluation worldwide. In total, over 500 clinical trials analyzing CAR-T cells for the treatment of cancer are currently being conducted around the world. Thereof, the majority are being performed in East Asia (269 trials), followed by the US (225 trials), and Europe with 62 ongoing studies (as registered at clinicaltrials.gov, Q3 2020).

Additionally, treatments with CAR-NK cells are becoming an alternative therapy option to CAR-T cells, as they possess certain advantages such as an intrinsic killing capacity of malignant cells and only few side effects post transplantation [19–22]. However, while a multitude of CAR-T cell therapies exist, still only relatively few CAR-NK cell trials are conducted worldwide. Currently, 19 trials investigating CAR-NK cells for the treatment of hematological malignancies as well as for the treatment of solid tumors are listed on clinicaltrials.gov. Most of the CAR-NK cell trials are conducted in China (15 trials), while three trials are ongoing in US, and only one trial is performed in Europe (Germany). In addition, a few trials are currently addressing CAR-NK/T cell products (2 trials in the US and 1 trial in China) as well as CAR-modified cytokine induced killer cells (1 trial in Italy).

## Current status of CAR-T and NK cell therapies in Germany

In Germany, 29 clinical trials are currently being conducted which evaluate CAR-modified immune cells, including two long-term follow-up (LTFU) studies. Furthermore, five studies have been completed or terminated to date (Table 1). Most of the trials investigate CAR-T cell products for the treatment of B-cell malignancies. But also other indications such as melanoma, acute myeloid leukemia (AML), and multiple myeloma (MM) are being evaluated. In addition, one ongoing CAR-NK cell study addresses the impact of CAR-NK92 cells in glioblastoma patients. In the following sections, a short description of the CAR-T and -NK cell trials conducted in Germany is provided. If not otherwise specified, information is derived from the European Clinical Trials Register clinicaltrialsregister.eu or from the US based trial register clinicaltrials.gov.

**Table 1:** Ongoing CAR-T and CAR-NK cell trials in Germany (listed on EU (clinicaltrialsregister.eu) and/or US Clinical Trials Register (clinicaltrials.gov)).

EudraCT (NCT)	Study title (acronym)									
	Status	Antigen	Disease	Phase	Start	No. Patients	Age	Vector	IMP	Sponsor
CD19-CAR-T cell products										
2018-000973-57^(o)^ (NCT03630159)	Phase Ib study of tisagenlecleucel in combination with pembrolizumab in r/r diffuse large B-cell lymphoma (DLBCL) patients (PORTIA)									
	O	CD19	DLBCL	1			>18	LV	Tisagenlecleucel	Novartis
2017-002116-14 (NCT03876769)	A phase II trial of tisagenlecleucel in first-line high-risk (HR) pediatric and young adult patients with B-cell acute lymphoblastic leukemia (B-ALL) who are minimal residual disease (MRD) positive at the end of consolidation (EOC) therapy (CASSIOPEIA)									
	O	CD19	B-ALL	2	2019	9	<18–64	LV	Tisagenlecleucel	Novartis
2017-004385-94 (NCT03568461)	A phase II, single arm, multicenter open-label trial to determine the efficacy and safety of tisagenlecleucel (CTL019) in adult patients with refractory or relapsed follicular lymphoma (ELARA)									
	O	CD19	FL	2	2019	12	>18	LV	Tisagenlecleucel	Novartis
2013-003205-25 (NCT02435849)	A phase II, single arm, multicenter trial to determine the efficacy and safety of CTL019 in pediatric patients with relapsed and refractory B-cell acute lymphoblastic leukemia (ELIANA)									
	O	CD19	B-ALL	2	2016	6	<18–64	LV	Tisagenlecleucel	Novarts
2014-003060-20 (NCT02445248)	A phase II, single arm, multicenter trial to determine the efficacy and safety of CTL019 in adult patients with relapsed or refractory diffuse large B-cell lymphoma (DLBCL) (JULIET)									
	O	CD19	DLBCL	2	2016	8	>18	LV	Tisagenlecleucel	Novartis
2016-002966-29 (NCT03570892)	Tisagenlecleucel versus standard of care in adult patients with relapsed or refractory aggressive B-cell non-Hodgkin lymphoma: A randomized, open label, phase III trial (BELINDA)									
	O	CD19	B-NHL	3	2019	37	>18	LV	Tisagenlecleucel	Novartis
2016-001991-31 (NCT03123939)	Phase IIIb study for relapsed/refractory pediatric/young adult acute lymphoblastic leukemia patients to be treated with CTL019									
	C	CD19	B-ALL	3	2017	5	<18–64	LV	Tisagenlecleucel	Novartis
2015-005007-86 (NCT02348216)	A phase I/II multicenter study evaluating the safety and efficacy of KTE-C19 in subjects with refractory aggressive Non-Hodgkin lymphoma (NHL) (ZUMA-1)									
	O	CD19	DLBCL, PMBCL, TFL	1/2	2017	60	18–64	RV	Axicabtagene ciloleucel	Kite
2017-002261-22 (NCT03391466)	A phase III, randomized, open-label study evaluating the efficacy of Axicabtagene Ciloleucel versus standard of care therapy in subjects with relapsed/refractory diffuse large B-cell lymphoma (ZUMA-7)									
	O	CD19	DLBCL	3	2018	48	>18	RV	Axicabtagene ciloleucel	Kite
2015-005010-30 (NCT02625480)	A phase I/II multicenter study evaluating the safety and efficacy of KTE-X19 in pediatric and adolescent subjects with relapsed/refractory B-precursor acute lymphoblastic leukemia or relapsed/refractory B-cell non-Hodgkin lymphoma (ZUMA-4)									
	O	CD19	B-ALL, B-NHL	1/2	2020	10	<18–64	RV	Brexucabtagene autoleucel	Kite
2018-001923-38 (NCT03624036)	Phase I/II multicenter study evaluating the safety and efficacy of KTE-X19 in adult subjects with relapsed/refractory chronic lymphocytic leukemia (ZUMA-8)									
	T	CD19	B-CLL	1/2	2019	15	>18	RV	Brexucabtagene autoleucel	Kite
2015-005009-35 (NCT02614066)	A phase I/II multicenter study evaluating the safety and efficacy of KTE-X19 in adult subjects with relapsed/refractory B-precursor acute lymphoblastic leukemia (r/r ALL) (ZUMA-3)									
	O	CD19	B-ALL	1/2	2018	5	18–64	RV	Brexucabtagene autoleucel	Kite
2015-005008-27 (NCT02601313)	A phase II multicenter study evaluating the efficacy of KTE-X19 in subjects with relapsed/refractory mantle cell lymphoma (r/r MCL) (ZUMA-2)									
	O	CD19	MCL	2	2018	20	>18	RV	Brexucabtagene autoleucel	Kite
2018-001246-34 (NCT03743246)	A phase I/II, open-label, single arm, multicohort, multicenter trial to evaluate the safety and efficacy of JCAR017 in pediatric subjects with relapsed/refractory B-ALL and B-NHL (TRANSCEND PEDALL)									
	O	CD19	B-ALL, B-NHL	1/2	2019	10	<18	LV	Lisocabtagene maraleucel	Celgene
2019-004081-18^(o)^ (NCT04245839)	A phase II, open-label, single-arm, multicohort, multicenter trial to evaluate the efficacy and safety of JCAR017 in adult subjects with relapsed or refractory indolent B-cell non-Hodgkin lymphoma (NHL) (TRANSCEND FL)									
	O	CD19	FL	2			>18	LV	Lisocabtagene maraleucel	Celgene
2017-000106-38 (NCT03484702)	A phase II, single-arm, multi-cohort, multicenter trial to determine the efficacy and safety of JCAR017 in adult subjects with aggressive B-cell non-Hodgkin lymphoma (TRANSCEND WORLD)									
	O	CD19	B-NHL	2	2018	16	>18	LV	Lisocabtagene maraleucel	Celgene
2018-000929-32^(o)^ (NCT03575351)	A global randomized multicenter phase III trial of JCAR017 compared to standard of care in adult subjects with high-risk, second-line, transplant-eligible relapsed or refractory aggressive B-cell non-Hodgkin lymphomas (TRANSFORM).									
	O	CD19	B-NHL	3			>18	LV	Lisocabtagene maraleucel	Celgene
2017-002848-32 (NCT03853616)	A phase I/II safety, dose finding and feasibility trial of MB-CART19.1 in patients with relapsed or refractory CD19 positive B-cell malignancies.									
	O	CD19	B-ALL, B-NHL, B-CLL	1/2	2018	48	<18, >18		MB-CART19.1	Miltenyi
2018-003916-38 (NCT04035434)	A phase I/II dose escalation and cohort expansion study of the safety and efficacy of allogeneic CRISPR-Cas9-engineered T cells (CTX110) in subjects with relapsed or refractory B-cell malignancies									
	O	CD19	B-NHL	1/2	2019	16	>18		CTX110	CRISPR
2016-004808-60 (NCT03676504)	Treatment of patients with relapsed or refractory CD19+ lymphoid disease with T-lymphocytes transduced by RV-SFG.CD19.CD28.4-1BBzeta retroviral vector—a unicenter Phase I /II clinical trial									
	O	CD19	B-ALL, B-NHL	1/2	2018	48	<18–64	RV	CD19.CAR T cells	UK HD
2007-007612-29 (NCT01195480)	Immunotherapy with CD19ζ chimeric antigen receptor gene-modified EBV-specific CTLs after stem cell transplant in children with high-risk acute lymphoblastic leukemia (CD19TPALL)									
	T^(*)^	CD19	B-ALL	1	2013	12	<18	RV	CD19 transduced EBV-CTL	UCL
CD19/CD20 -Dual-CAR-T cell products										
2018-001253-27^(o)^ (NCT03870945)	A phase I/II safety, dose finding and feasibility trial of MB-CART2019.1 in patients with relapsed or resistant CD20 and CD19 positive B-NHL									
	O	CD19, CD20	B-NHL	1/2			>18	LV	MB-CART2019.1	Miltenyi
CD20 -CAR-T cell products										
2017-000120-10^(o)^ (NCT03893019)	Multicenter phase I trial of MB-CART20.1 for the treatment of patients with metastatic melanoma									
	O	CD20	Melanoma	1			>18	LV	MB-CART20.1	Miltenyi
2017-000121-12^(o)^ (NCT03664635)	A phase I/II safety, dose finding and feasibility trial of MB-CART20.1 in patients with relapsed or resistant CD20-positive B-NHL									
	O	CD20	B-NHL	1/2			>18	LV	MB-CART20.1	Miltenyi
CD123-CAR-T cell products										
2019-001339-30^(o)^ (NCT04230265)	Multicenter, open-label, adaptive design phase I trial with genetically modified T cells carrying universal chimeric antigen receptors (UniCAR02-T) in combination with CD123 Target Module (TM123) for the treatment of patients with hematologic and lymphatic malignancies positive for CD123									
	O	CD123	AML, B-ALL, BPDCN	1			>18	LV	UniCAR02-T + TM123	Cellex
BCMA-CAR-T cell products										
2018-000264-28 (NCT03601078)	A phase II, multi-cohort, open-label, multicenter study to evaluate the efficacy, and safety of bb2121 in subjects with relapsed and refractory multiple myeloma and in subjects with clinical high-risk multiple myeloma (KarMMa-2)									
	O	BCMA	MM	2	2019	10	>18	LV	Idecabtagene vicleucel	Celgene
2017-002245-29 (NCT03361748)	A phase 2, multicenter study to determine the efficacy and safety of bb2121 in subjects with relapsed and refractory multiple myeloma (KarMMa)									
	O	BCMA	MM	2	2018	15	>18	LV	Idecabtagene vicleucel	Celgene
2018-001023-38 (NCT03651128)	A phase III, multicenter, randomized, open-label study to compare the efficacy and safety of BB2121 versus standard regimens in subjects with relapsed and refractory multiple myeloma (RRMM) (KarMMa-3)									
	O	BCMA	MM	3	2019	40	>18	LV	Idecabtagene vicleucel	Celgene
2018-004124-10^(o)^ (NCT04133636)	A phase 2, multicohort open-label study of JNJ-68284528, a chimeric antigen receptor T cell (CAR-T) therapy directed against BCMA in subjects with multiple myeloma (CARTITUDE-2)									
	O	BCMA	MM	2			>18	LV	JNJ-68284528	Janssen
SLAMF7-CAR-T cell product										
2019-001264-30 (NCT04499339)	A phase I/IIa clinical trial to assess feasibility, safety and antitumor activity of autologous SLAMF7 CAR-T cells in multiple myeloma									
	O	SLAMF7	MM	1/2	2020	12	>18	SB	SLAMF7 CAR-T	UK Würzburg
CLDN6-CAR-T cell product										
2019-004323-20 (NCT04503278)	Phase I/IIa, first-in-human, open-label, dose-escalation trial with expansion cohorts to evaluate safety, and preliminary efficacy of CLDN6-CAR-T with or without CLDN6 RNA-LPX in patients with CLDN6-positive relapsed or refractory advanced solid tumors									
	O	Claudin-6	Solid tumor	1/2	2020	18	>18	RV	CLDN6-CAR-T	BioNTech
HER2-CAR-NK-cell product										
2016-000225-39^(o)^ (NCT03383978)	Multicenter, open label, phase I study of intracranial injection of NK-92/5.28.z cells in patients with recurrent HER2-positive glioblastoma (CAR2BRAIN)									
	O	Her2	Glioblastoma	1			>18	LV	NK-92/5.28.z cells	KGU
Long-term follow-up studies										
2017-001465-24 (NCT03435796)	Long-term follow-up protocol for subjects treated with gene-modified T cells.									
	O				2018	55				Celgene
2014-001673-14 (NCT02445222)	Long-term follow-up of patients exposed to lentiviral-based CAR T cell therapy (PAVO)									
	O				2016	55				Novartis

Trials are subdivided depending on the target antigen, investigational medicinal product (IMP), phase and start date of study (in chronological order). In addition, the study title, study acronym, status of the study in Germany, disease for which the drug is tested, intended number of trial participants in Germany, age of participants, vector which was used to transduce cells, and the sponsor of the study is listed.

*Status:* O ongoing, *T* terminated, *C* completed. *Vector*: *LV* lentiviral vector, *RV* retroviral vector, *SB* sleeping beauty. *Disease:* *ALL* acute lymphoblastic leukemia, *BPDCN* blastic plasmacytoid dendritic cell neoplasm, *CLL* chronic lymphocytic leukemia, *DLBCL* diffuse large B-cell lymphoma, *FL* follicular lymphoma, *IMP* investigational medicinal product, *MCL* mantel cell lymphoma, *MM* multiple myeloma, *NHL* Non-Hodgkin Lymphoma, *PMBCL* primary mediastinal B-cell lymphoma, *TFL* transformed follicular lymphoma, *r/r* relapsed/refractory. *Sponsor:* *Celgene* Celgene Corporation, *Cellex* Cellex Patient Treatment GmbH, *KGU* Johann Wolfgang Goethe University Hospital, *Kite* Kite Pharma, Inc., *Miltenyi* Miltenyi Biomedicine GmbH and Miltenyi Biotec B.V. & Co. KG Novartis, Novartis Pharma.

^(o)^Complete data set derived from clinicaltrials.gov.

^(*)^Indicated data derived from clinicaltirals.gov.

## CAR-T cell therapies

### CAR-T cells to target B-cell malignancies

The human CD19 antigen is a 95 kd transmembrane glycoprotein which belongs to the immunoglobulin superfamily without any known homology for other proteins [23]. CD19 positive cancers include diseases with high-priority medical needs such as r/r B-cell acute lymphoblastic leukemia (B-ALL), B-cell chronic lymphocytic leukemia (B-CLL), B-cell Non-Hodgkin lymphoma (B-NHL), and other B-cell malignancies. As CD19 is a B-cell-specific surface protein that is expressed throughout B-cell development, it is present on most B-cell malignancies and therefore a suitable target for CAR-T cell therapies [24]. Thus, in most clinical cell therapy trials for treatment of B-cell malignancies, CAR-T cells are used which are engineered to express CD19-specifc CARs. In addition to that, two studies are being conducted which deploy T cells expressing either CD20-CARs or CD19/CD20-dual-CARs.

#### Kymriah

The approval of *Tisagenlecleucel* (market name Kymriah^®^; lab code CTL019 or tisa-cel; producer: Novartis AG) in August 2017 by the FDA as first-in-class therapy set a milestone in cancer therapy [25]. Nearly 1 year later, in August 2018, Kymriah also received approval in Europe by the EC [26, 27].

Kymriah is generated from autologous CD4/CD8 T-cell enrichment of peripheral blood mononuclear cells (PBMCs) which are further transduced using a lentiviral vector. The vector encodes a second-generation CAR harboring the scFv, derived from the CD19-specific monoclonal antibody FMC63 and the co-stimulatory domain from 4-1BB in conjunction with the signaling domain from CD3zeta [23, 26].

Kymriah is applied as therapy for patients up to 25 years with r/r B-ALL and adult patients with r/r large B-cell lymphomas after two or more lines of systemic therapies. Treatments with Kymriah have already achieved striking results with initial complete-response rates of more than 80% for the indication r/r B-ALL [28, 29]. Results for the treatment of diffuse large B-cell lymphoma (DLBCL) demonstrated a higher response durability compared to the historical control [26, 28]. At the moment, seven clinical trials along with one LTFU study of Kymriah are being conducted in Germany.

The approval of Kymriah is based on the phase II, single arm, multicenter study ELIANA (NCT02435849) for the indication B-ALL and on the phase II open-label, multicenter, single-arm study JULIET (NCT02445248) for the indication DLBCL [26], which were conducted in Germany as well. Clinical trial sites were located in Frankfurt and Cologne (ELIANA) and Würzburg (JULIET).

To further collect data on the efficacy of Kymriah in a B-NHL setting, the post-authorization study BELINDA (NCT03570892), a phase 3, randomized, controlled trial of Kymriah versus standard-of-care chemotherapy is currently performed across the US, Australia, Germany, Japan, and Spain with a target enrollment of 318 patients. Seven different trial sites participate in Germany (e.g., Regensburg, Berlin, Hamburg) and intend to include 37 patients.

Another phase III multicenter study is currently ongoing in several different countries in Europe as well as in Canada and Japan treating children and young adults suffering from B-ALL (NCT03123939). In Germany, the trial is already completed. In total, five patients were intended to be treated at the clinical site in Frankfurt. Outcomes in this study remain mostly consistent with those in ELIANA [30].

At the moment, two further multinational, multicenter, phase II studies with Kymriah are ongoing in Germany investigating its use for adult patients suffering from r/r follicular lymphoma (FL) and the feasibility to treat first-line high-risk pediatric and young adult patients with B-ALL at the end of consolidation therapy (ELARA, NCT03568461 and CASSIOPEIA, NCT03876769). In total 12 and 9 patients, respectively, are intended to be treated in Germany. Participating study sites in Germany are located in Cologne, Ulm and Munich for ELARA as well as Frankfurt and Munich for CASSIOPEIA.

In addition, a phase I multicenter study is currently conducted in Cologne, Germany, as well as in Austria, the US and Canada, testing Kymriah in combination with the PD-1 inhibitor Pembrolizumab in a cohort of up to 32 patients suffering from DLBCL (PORTIA, NCT03630159). First results of 4 treated patients showed a manageable safety profile of Pembrolizumab in combination with Kymriah without dose-limiting toxicities or clinical significant exacerbations of adverse events [31].

The LTFU study PAVO (NCT02445222) aims to evaluate the risk of delayed adverse events, check for replication competent lentivirus and estimate long-term efficacy, including CAR-T cell persistence. In this non-interventional study, Novartis monitors up to 1,250 patients for 15 years, who had been exposed to one of the CAR-T cell therapies using Kymriah.

In addition to the above-mentioned trials, 12 ongoing studies investigate Kyrmiah outside Germany.

#### Yescarta

The second approved CAR-T cell product to date, is *Axicabtagene Ciloleucel* (market name Yescarta^®^; lab code KTE-C19 or axi-cel; producer: Kite, a Gilead Science, Inc company). It has received marketing authorization in Europe parallel to Kymriah in August 2018 and just a few months later in the US (October 2018) [32, 33]. For this therapy, patient-derived T cells are transduced using a gamma-retroviral vector that expresses a second-generation CAR which targets CD19 similar to Kymriah. Thus it possesses a different intracellular co-stimulatory domain derived from CD28 [34]. Notably, Yescarta is generated from CD3^+^ enriched autologous T cells.

Yescarta is authorized as therapy for adult patients with r/r DLBCL and primary mediastinal large B-cell lymphoma, after two or more lines of systemic therapy. The key study for approval, partly being conducted in Germany, was the phase II part of the phase I/II multicenter study ZUMA-1 (NCT02348216). In this still ongoing study, the safety and efficacy of Yescarta has been analyzed and the effects of prophylactic regimens or earlier interventions on the abundance and severity of adverse side effects has been evaluated. At the time of marketing authorization application (MAA), data from 111 patients across 24 trial sites were available, demonstrating a higher overall response rate for patients treated with Yescarta compared to the historical control [32, 35, 36]. In total, 307 adult patients are planned to be included, at 37 study locations. Out of these, up to 60 patients are treated in Germany at the university hospitals of Dresden, Essen and Würzburg. Further real-world safety and efficacy data on the use of Yescarta is expected through the expanded access trial ZUMA-9 (NCT03153462) conducted in the US only.

Currently, one further study investigating Yescarta is conducted in Germany. This study is a phase III multicenter study in which Yescarta is compared to standard care therapy in up to 359 patients with r/r DLBCL at 77 different study locations (ZUMA-7, NCT03391466). Thereof, 48 patients are planned to be treated at six different study sites in Germany, including the university hospitals of Dresden, Göttingen and Hamburg-Eppendorf.

In addition to the above-mentioned trials, currently 13 ongoing studies are evaluating treatment protocols with Yescarta outside Germany.

#### Tecartus

At the beginning of 2020, Kite announced the MAA for its second CAR-T cell product *brexucabtagene autoleucel* (market name Tecartus™; lab code KTE-X19 or brexu-cel), which has been currently approved by the FDA (July 2020) and the EC (December 2020) [37, 38]. Notably, Tecartus is a T-cell product similar to Yescarta in terms of generation and CAR structure, but is the first and only CAR-T cell therapeutic for adult patients suffering from r/r mantle cell lymphoma (MCL) [39–42]. The clinical safety review by the FDA was primarily based on the analysis of 88 patients that have been treated in the ZUMA-2 study (NCT02601313) [39], which is an ongoing phase II multicenter study for the treatment of r/r MCL conducted at various sites in the US and Europe with up to 105 patients in total. In Germany, up to 20 patients are treated at the university hospitals of Dresden and Würzburg. The ZUMA-2 clinical trial has already generated impressive results, as 93% of the patients responded to a single infusion of Tecartus and 67% attained a complete response [38, 40].

Furthermore, Tecartus is currently being tested in four different studies, partly located in Germany, not only for the indication MCL but also for the treatment of B-ALL or B-NHL, including chronic lymphocytic leukemia (CLL). In the two phase I/II multicenter studies ZUMA-3 and ZUMA-4 (NCT02614066, NCT02625480) over 100 patients, each suffering from r/r B-ALL or B-NHL will be treated with Tecartus. The study locations are distributed all over the US and Europe. In Germany, five (ZUMA-3) and ten patients (ZUMA-4) are intended to be treated in at least three different study sites (Frankfurt, Munich, Würzburg).

Moreover, Tecartus is also being tested for patients with r/r B-CLL in one phase I/II multicenter study (ZUMA-8, NCT03624036). It is currently ongoing at 21 different study locations in the US. It was planned to conduct the trial in Germany as well, but it was prematurely ended.

#### JCAR017

Celgene, as part of the US biotech company Bristol-Myers Squibb™, initiated its first clinical trial in Germany in 2016 with the second-generation CAR-T cell product JCAR015 (NCT02973191). However, this study was withdrawn owing to the development of cerebral edema and subsequent death of several B-ALL-patients, who had participated in the phase II ROCKET trial (NCT02535364) [43–47]. Subsequently, Celgene proceeded with JCAR017 as therapy for r/r DLBCL, another CAR-T cell product, which possesses an improved safety profile compared to JCAR015 [43, 46]. While cells used to create JCAR015 were composed of CD3^+^ enriched PBMCs, which were transduced using a gamma-retroviral vector, in JCAR017, a fixed ratio of CD4^+^ and CD8^+^ cells (1:1) is transduced using lentiviral vectors [48]. Additionally, Celgene changed the CD19-specific scFv-based binding domain and co-stimulatory domain from SJ25C1 to FMC63 and CD28 to 4-1BB in JCAR017, respectively. Furthermore, they included a truncated version of the human epidermal growth factor receptor (EGFRt) in JCAR017, which might offer the potential to induce killing of CAR-T cells following addition of cetuximab (Erbitux^®^) [43, 46]. Taking these changes into consideration, their new product JCAR017 already showed promising results in their TRANSCEND NHL 001 trial in patients with r/r pediatric B-ALL and B-NHL (NCT02631044) [48]. Based on these findings, Bristol-Myers Squibb recently submitted the product JCAR017 (international nonproprietary name *Lisocabtagene maraleucel;* market name Breyanzi^(R)^ lab code liso-cel) for authorization in the US and Europe [49, 50] and just recieved approval in the US [18].

Currently, there are nine ongoing clinical trials worldwide which use JCAR017. Of these, four are conducted in Germany. The most advanced trial is the multicenter phase III study TRANSFORM, that will include 182 adult patients with high-risk, aggressive r/r B-NHL (NCT03575351). Patients are being treated at 53 study locations in the US and Europe including six study sites in Germany, e.g., the university hospitals of Dresden, Cologne and Munich. Additionally, JCAR017 is deployed in the multicenter phase II study TRANSCEND WORLD, which will contain 116 participants suffering from similar diseases (NCT03484702). This study is being conducted at 19 different study sites across Europe with 16 patients scheduled to be treated in five centers in Germany, such as the university hospitals of Heidelberg, Cologne and Munich.

In the phase II multicenter study TRANSCEND FL, JCAR017 will be tested in 188 patients with r/r indolent B-NHL (NCT04245839). Patients will be treated in 36 study locations all around the world including study sites in Germany Cologne, Munich and Ulm. Furthermore, one phase I/II multicenter study with JCAR017 is being conducted (NCT03743246). This study will comprise 121 pediatric patients suffering from r/r B-ALL and B-NHL. Out of these patients, ten are going to be treated in Germany.

Similar to Novartis, Celgene is also conducting a LTFU study with patients who had received a gene-modified `T cell therapy sponsored by Celgene or alliance partners (NCT03435796). In this study, patients will be monitored over 15 years for long-term safety and efficacy following the last CAR-T cell infusion.

#### MB-CART19.1

In the field of CD19^+^ malignancies the German company *Miltenyi Biotec* has also developed a CD19-targeting T-cell product called *MB-CART19.1*. It is generated by transduction of autologous CD4^+^/CD8^+^ enriched T cells using a lentiviral vector encoding a second-generation CAR which harbors 4-1BB as co-stimulatory domain [51, 52]. MB-CART.19.1 is currently being tested in one clinical trial in Germany only. This phase I/II multicenter study will include up to 48 pediatric and adult participants suffering from r/r CD19^+^ B-cell malignancies such as B-ALL, B-CLL, and B-NHL (NCT03853616) at the sites Erlangen and Muenster.

#### CTX110

While most companies use autologous enriched T cells to avoid graft-versus-host disease (GvHD) and viral vectors for transduction, some companies envision new approaches such as the Swiss company *CRISPR Therapeutics* that is specialized in gene editing using the clustered regularly interspaced short palindromic repeats (CRISPR)/Cas9 technology.

*CRISPR Therapeutics’* T-cell product *CTX110* consists of allogeneic derived T cells which are ex vivo edited by use of the CRISPR/Cas9 technology to insert the CAR-construct precisely into the TCR alpha constant (TRAC) locus and simultaneously disrupt endogenous T-cell expression to reduce the risk of GvHD. In addition, to improve CAR-T cell persistence, major histocompatibility complex expression is eliminated to avoid rejection of the allogeneic CAR-T cell product by the patient’s own immune cells [53]. The idea behind this concept is to generate off-the-shelf CAR-T cell products which is very appealing to circumvent hurdles associated with the complex manufacturing process of autologous CAR-T cell therapies [11]. Currently, *CTX110* is being tested in one phase I/II multicenter study with 95 patients suffering from r/r B-cell malignancies such as B-NHL or B-cell lymphoma (NCT04035434). Out of these patients, 16 are planned to be treated in Hamburg, Germany.

Besides CTX110, CRISPR *Therapeutics* currently also develops allogeneic CAR-T cell product for the treatment of r/r MM (CTX120, NCT04244656) and solid tumors (CTX130, NCT04438083).

#### Other CD19-specific CAR-T cell products

Not only do companies draft clinical trials using CD19-targeting CAR-T cell therapies, but also investigator-driven clinical studies are performed, such as the *CD19.CAR T* trial at the university hospital of Heidelberg. This trial deploys autologous T cells which are retrovirally transduced to express a third generation CAR which possesses the intracellular co-stimulatory domains derived from CD28 and 4-1BB. In this phase I/II study, 48 adult patients suffering from r/r B-ALL, B-NHL, B-CLL, DLBCL, FL, or MCL will be treated with escalating doses of *CD19.CAR T cells* (NCT03676504). It has been reported so far that patients treated with *CD19.CAR T cells* responded clinically, despite the administration of low cell numbers. Injected CAR-T cells showed a very good safety profile, were detectable in the body for over 3 months post administration and were able to migrate into different compartments [54].

Furthermore, a clinical trial evaluating the feasibility, safety and biological effect of adoptive transfer of donor-derived EBV-specific cytotoxic T-lymphocytes (EBV-CTL) transduced with a gamma-retroviral vector encoding a first generation CD19-specific CAR construct for the treatment of patients with high-risk or relapsed B-cell precursor ALL after allogeneic hematopoietic stem cell transplantation was conducted in Germany and the UK (NCT01195480). This trial intended to include up to 12 patients in Germany but was terminated due to the lack of biological efficacy and CAR-T cell persistence as reported at clinicaltrials.gov.

#### MB-CART20.1 and MB-CART2019.1

In order to treat B-cell malignancies, not only CD19 but also CD20 seems to be a very suitable target, as it is tightly restricted and expressed on most B cells starting early in the pre-B stage of B-cell ontogeny. Owing to the fact that it is absent on precursor lymphoid cells and majority of plasma cells, killing of CD20^+^ cells should not disrupt monoclonal antibody (mAb) production and also maintain B-cell regeneration post therapy [55, 56].

Hence, *Miltenyi Biotec* developed two different CD20-targeting T-cell products, generated from autologous CD4^+^/CD8^+^ enriched T cells transduced, using a lentiviral vector. One is called *MB-CART20.1 and* consists of T cells expressing a second-generation CD20-specific CAR harboring the 4-1BB co-stimulatory domain. This product is currently being tested in two studies exclusively in Germany. In the phase I/II dose-finding study, 19 patients suffering from CD20^+^ r/r B-NHL are intended to be included at the university hospitals of Cologne and Leipzig (NCT03664635). The second trial investigates the usage of MB-CART20.1 for the treatment of unresectable stage III or IV melanoma in up to 15 patients in an earl phase I multicenter study at the University Hospital Cologne (NCT03893019). Notably, this is the first trial with CD20-CAR transduced T cells against melanoma conducted in Europe and is based on findings that melanoma cancer sustaining cells express CD20 [57, 58].

The other T cell product by *Miltenyi Biotec*, *MB-CART2019.1*, comprises T cells engineered to express tandem CAR constructs targeting both CD19 and CD20. This approach offers the potential to prevent emergence of antigen escape and was shown to be more effective and less toxic in a higher disease burden setting in a preclinical model [59, 60]. Currently *MB-CART2019.1* is being tested in one multicenter phase I/II dose-finding trial in Germany with up to 12 adult patients suffering from r/r, aggressive CD19- and CD20-positive B-NHL/CLL or B-cell small lymphocytic lymphoma (B-SLL) (NCT03870945). Study sites include the university hospitals of Cologne, Hamburg-Eppendorf and Augsburg.

### CAR-T cells targeting AML

While most CAR-T cell therapies are designed to target B-cell malignancies, they are also under development for the treatment of different hematologic diseases such as AML. Besides CD33, which was detected on AML blasts in 85–90% of patients and shown to be present on leukemic stem cells (LCSs), also CD123 expression could be detected on AML blasts as well as LCSs in 75–89% of cases [61–64]. Thus, both antigens were evaluated as promising targets for AML-specific CAR-cellular therapies. In line with these findings, the German company *Cellex Patient Treatment GmbH* created a modular universal CAR platform called UniCAR, to generate adapter CARs which consists of two components: a targeting module (TM)-specific CAR for the initial T-cell engineering and a TM which redirects the Uni-CAR-T cells to a specific target (e.g., a CD33 or CD123 antigen on AML blasts). This system was designed to easily change targets of CAR-T cells by simply replacing the TMs. Applications include targeting of more than one antigen simultaneously, consecutive addition of different TMs to enhance therapy efficacy and to reduce the risk of antigen-loss tumor variants during the treatment [65–67].

Currently the study drug UniCAR02-T-CD123 is tested for its safety and efficacy in one dose-escalating multicenter phase I trial (NCT04230265) which is conducted in Germany. UniCAR02-T-CD123 consist of the cellular component UniCAR02-T and a recombinant antibody derivative targeting CD123 (TM123). The study will comprise 45 participants with hematologic and lymphatic malignancies such as AML, B-ALL, and blastic plasmacytoid dendritic cell neoplasm positive for CD123. The patients will be treated in five study sites in Germany, including the university hospitals of Würzburg and Dresden.

### CAR-T cells for the treatment of multiple myeloma

Another disease with unmet medical needs is MM. It is a progressive, usually incurable disease, probably resulting from multiple genetic mutations to the precursor plasma cell. All MM cells express the B-cell maturation antigen (BCMA), a transmembrane glycoprotein in the TNF receptor superfamily 17 (TNFRSF17), which is not expressed on other normal tissues except on normal plasma cells and was demonstrated to be a suitable target for CAR-T cell therapy [68]. In this line, BCMA-specific CAR-T cells have demonstrated clinical response in patients with r/r MM, that have undergone at least three prior treatments [69].

The most advanced BCMA-CAR-T cell product for the treatment of r/r MM is *idecabtagene vicleucel* (lab code ide-cel, bb2121), an investigational BCMA-specific CAR-T cell therapy developed by the US pharma company *Brystol Myers Squibb* (former company: *Celgene*), which is currently under evaluation in the US and Europe to receive marketing authorization [70, 71]. Ide-cel consists of autologous T cells transduced with a lentiviral vector to express a BCMA-specific second-generation CAR, which possess 4-1BB as intracellular co-stimulatory domain [72, 73]. The MAA is based on the results derived from the pivotal phase 2 KarMMa study evaluating the efficacy and safety of ide-cel in heavily pre-treated patients with relapsed and refractory MM (NCT03361748) [71]. In this ongoing study up to 149 patients at 24 study locations worldwide will be investigated. Out of these, 15 patients are scheduled to be treated in Germany at the university hospitals of Würzburg, Heidelberg and Tübingen. Initial results of 128 treated patients demonstrated deep, durable responses of ide-cel with an overall response rate of 73% and a favorable clinical benefit-risk profile across the target dose range [74].

Furthermore, ide-cel is currently being tested in five different studies for the treatment of r/r or high-risk MM which are partly located in Germany. In the KarMMa-2 study (NCT03601078), a phase II multicenter trial evaluating the safety and efficacy of ide-cel in high-risk MM patients, up to 181 participants will be included across 26 study locations in the US and Europe. In Germany ten patients are scheduled to be treated at the university hospitals of Würzburg, Hamburg and Tübingen. In the phase III multicenter trial KarMMA-3 ide-cel will be compared to standard of care treatment in up to 381 participants at 49 different study sites located all over the world (NCT03651128). Thereof, 40 patients are planned to be treated in Germany at the university hospitals of Würzburg, Heidelberg and Cologne.

In addition to *Brystol Myers Squibbs’* ide-cel, also the US pharma company *Janssen Pharmaceutica* (part of *Johnson & Johnson*) is testing the BCMA-targeting CAR-T cell therapeutic JNJ-68284528 in Germany. CAR-T cells in JNJ-68284528 possess two BCMA-targeting single-domain antibodies designed to enable avidity of the CAR construct towards BCMA [75]. In its phase II trial CARTITUDE-2, JNJ-68284528 is going to be applied to 100 patients suffering from MM (NCT04133636). The trial sites are located in the US, Israel and Europe and include the University hospitals of Würzburg, Heidelberg and Hamburg-Eppendorf in Germany. Notably, the efficacy of JNJ-68284528 is currently addressed in two more trials for the treatment of MM without the contribution of German trial sites.

Another antigen which is currently under evaluation for CAR-T cell therapy in patients with MM is SLAMF7 (signaling lymphocytic activation molecule F7, also known as CS1). SLAMF7 was shown to be highly expressed on malignant plasma cells in MM and has functional significance [76]. In the ongoing phase I/II clinical trial CARAMBA-1 (NCT04499339) conducted by the University Hospital Würzburg, the safety, feasibility and antitumor activity of autologous SLAMF7-specific CAR-T cells is tested in up to 38 patients suffering from MM. The patients will be treated at the trial sites Würzburg, Germany; Pamplona, Spain; Milano, Italy and Lile, France. SLAMF7-specific CAR-T cells in this study are generated by the Sleeping Beauty Transposon gene transfer. This constitutes an alternative virus-free method with the aim to increase the safety and reduce the production costs of manufactured CAR-T cells [77]. This project started in January 2018 and is funded by the EU Horizon 2020 program [78, 79].

### Claudin-6 (CLDN6) CAR-T cells to fight solid tumors

Although CAR-T cells appear to be very effective in targeting hematological diseases, many challenges still exist for the usage of CAR-T cells to treat solid tumors. These are for instance the immune-suppressive effects of the tumor microenvironment, the trafficking of CAR-T cells to the tumor site and tumor infiltration as well as the lack of cancer-specific solid tumor targets to reduce severe on-target/off-tumor toxicities [80].

Nevertheless, academia and companies are trying to employ CAR-T cells for the treatment of solid tumors such as the German company *BioNTech Cell & Gene Therapies GmbH*. Their CAR-T cell product BNT211 consists of autologous T cells equipped with a second-generation CAR specific for the tight junction protein Claudin-6 (CLDN6) that will be administered in combination with a CAR-T cell amplifying RNA Vaccine (CARVac) to drive expansion, persistence and efficacy of the CLDN6-CAR-T cells against solid tumors [81, 82]. CARVac is derived from *BioNTech´s* proprietary RNA lipoplex (RNA-LPX) technology tailor-made for body-wide delivery of antigen to lymphatic tissue upon systemic administration [83]. Importantly, RNA-LPX is being used in numerous clinical trials for induction of endogenous T-cell responses against a plethora of cancer antigens (e.g., NCT02410733, NCT02316457, and NCT03815058). In its first phase I/II multicenter cell therapy study, *BioNTech* wants to evaluate the safety and efficacy of BNT211 in patients with CLDN6^+^ r/r advanced solid tumors such as ovarian, testicular, uterine, lung and gastric cancer (NCT04503278).

## CAR-NK cell therapies

NK cells have recently moved into the spotlight as an imposing tool for immunotherapy. While NK cells utilize similar killing mechanisms for eliminating malignant or virally infected cells compared to cytotoxic T-lymphocytes (CTLs), their target recognition mechanism differs substantially. CTLs as part of the adaptive immune response, recognize their targets via a vast variety of clonally rearranged T-cell receptors (TCRs). NK cells on the other hand, as innate lymphoid cells, receive activating and inhibitory signals by their germline-encoded receptor repertoire [84]. The ability to recognize the absence of HLA-proteins, specializes NK cells to identify malignant or virally infected cells, which often downregulate these proteins to escape immune surveillance [22]. Thus, a CAR-NK cell therapy could offer the advantage of an intrinsic tumor killing capacity possessed by CAR-NK cells in addition to their CAR-depended killing mechanism, impeding tumor immune escape mechanisms. Furthermore, the absence of TCRs strongly reduces the risk of GvHD, which potentially could enable allogeneic CAR-NK cell transplantations [19–21, 85]. The latter could reduce the tremendous costs required as well as the limited availability of an autologous therapy, which is caused by logistics and the low cell numbers of often heavily pre-treated patients [11, 86].

CAR-NK cell therapies have been evaluated lately in different tumor settings. Quite recently, the group of Katy Rezvani published clinical results of the first 11 patients receiving CAR-NK cells derived from umbilical cord blood (UCB) for targeting CD19-expressing B-cell malignancies (NCT03056339). Eight out of these 11 patients showed a clinical response (73%). Of these patients, seven had complete remission and the responses were rapid [86]. This and other trials indicate that CAR-NK cells might represent a promising therapeutic option with the above-mentioned beneficial properties.

### HER2-specifc CAR-NK cells for the treatment of glioblastoma

Therapies involving primary NK cell preparations are faced with hurdles, such as the challenging viral transduction, and the still cost- and time-intensive expansion of primary NK cells under good manufacturing practice (GMP) conditions [87]. Hence, the first and currently only ongoing clinical trial with CAR-NK cells in Germany deploys glioblastoma-specific CAR-NK-92 cells (CAR2BRAIN, NCT03383978) [88]. NK-92 is the only human NK cell line to date approved for the treatment of patients in clinical trials [89, 90]. NK-92 cells hold the advantage of being easily expandable under GMP conditions, and display features of activated NK cells with high antitumor efficacy due to the lack of most inhibitory NK cell receptors [89, 91, 92]. Nevertheless, NK-92 cells also face critical obstacles such as the requirement for irradiation to reduce their proliferative capacity, as they are derived from a lymphoma [93]. Additionally, NK-92 cells are IL-2 dependent, which could result in toxicities if repeated IL-2 injections are combined with cellular therapy [93–96]. In the phase I clinical trial CAR2BRAIN, intrathecal administration of genetically engineered NK-92/5.28.z cells during relapse surgery is being tested at the University Hospital Frankfurt in patients suffering from recurrent HER2^+^ glioblastoma (GB) [88]. This cell product consists of lentivirally transduced NK-92 cells expressing a human epidermal growth factor 2 (HER2, ErbB2)-specific second-generation CAR, which harbors a composite CD3ζ-CD28 signaling moiety [97]. The single-dose dose-escalation part of the study has recently been completed, without encountering dose-limiting toxicities at the three applied dose levels. Currently, patients of the expansion cohort of the trial are being recruited, scheduled to receive repeated weekly injections of irradiated NK-92/5.28.z cells into the resection cavity through an implanted catheter and reservoir [88]. HER2 was chosen as target, as it was found to be expressed by about 40% of primary GB cells and the majority of GB cell lines. An effective therapy is urgently needed, as GBM is the most common and aggressive primary brain cancer, with low 5-year overall survival rates following standard of care therapy including surgical resection and adjuvant chemotherapy [97, 98].

## Future perspective of CAR-T and CAR-NK studies

The field of CAR-cellular therapies is still young, but preclinical and clinical studies have already shown remarkable results. To date the first four drugs in the US and three in Europe have so far been granted marketing authorization, using CAR-modified immune cells as therapies for specific hematological malignancies. Thus, a multitude of targets have yet to be addressed, with even considering nonmalignant targets such as HIV-infected cells [99, 100]. In this context, it is important to keep in mind that adjustments can be made regarding the CAR structure, additional genetic modifications and the choice of the adoptive immune cells. The combination of cellular therapies with other drugs such as immune-checkpoint inhibitors could further improve clinical outcomes. Therefore, we believe that the field of CAR-based cellular therapies is profoundly promising and is certainly going to lead to the development of approved personalized therapeutic options in the future.
